# Epithelial–Mesenchymal Plasticity and Epigenetic Heterogeneity in Cancer

**DOI:** 10.3390/cancers16193289

**Published:** 2024-09-27

**Authors:** Jessica L. Sacco, Esther W. Gomez

**Affiliations:** 1 Department of Chemical Engineering, The Pennsylvania State University, University Park, PA 16802, USA; jls7903@psu.edu; 2 Department of Biomedical Engineering, The Pennsylvania State University, University Park, PA 16802, USA

**Keywords:** epithelial–mesenchymal transition, phenotype, epigenetic, matrix stiffness, mechanical stress, intratumoral heterogeneity

## Abstract

**Simple Summary:**

Phenotypic diversity in tumors makes treatment difficult and leads to poor prognosis. A better understanding of what causes intratumoral heterogeneity and differences in cells that are within the same tumor will advance therapeutic options and improve patient outcomes. This review will summarize recent studies that have examined the various cancer cell phenotypes along the epithelial–mesenchymal spectrum present in tumors and how epigenetic factors and the mechanical properties of tumor tissue mediate these phenotypes. This review will also discuss the therapeutic implications of intratumoral heterogeneity, such as drug resistance and tumor recurrence, and highlight opportunities to address current limitations in cancer treatments.

**Abstract:**

The tumor microenvironment comprises various cell types and experiences dynamic alterations in physical and mechanical properties as cancer progresses. Intratumoral heterogeneity is associated with poor prognosis and poses therapeutic challenges, and recent studies have begun to identify the cellular mechanisms that contribute to phenotypic diversity within tumors. This review will describe epithelial–mesenchymal (E/M) plasticity and its contribution to phenotypic heterogeneity in tumors as well as how epigenetic factors, such as histone modifications, histone modifying enzymes, DNA methylation, and chromatin remodeling, regulate and maintain E/M phenotypes. This review will also report how mechanical properties vary across tumors and regulate epigenetic modifications and E/M plasticity. Finally, it highlights how intratumoral heterogeneity impacts therapeutic efficacy and provides potential therapeutic targets to improve cancer treatments.

## 1. Introduction

The tumor microenvironment consists of various cell types including cancer cells, immune cells, endothelial cells, and stromal cells such as cancer-associated fibroblasts [1]. Single-cell analysis of human tumor samples has revealed vast phenotypic diversity of tumor and immune cells and identified subpopulations of cells with phenotypes associated with poor prognosis and immunosuppression [2]. The phenotypic diversity of cancer cells within a tumor, often referred to as intratumoral heterogeneity, can contribute to drug resistance [3]. Indeed, a high degree of intratumoral heterogeneity is associated with poor survival in multiple cancers including colorectal, head and neck squamous cell, breast, kidney, prostate, melanoma, and glioma [4,5].

Phenotypic diversity in cancer cells can be mediated by epithelial–mesenchymal transition (EMT). EMT is a cellular process where cells experience alterations in phenotype, switching from epithelial to mesenchymal. During EMT, cells lose cell–cell contacts, gain motility, and undergo significant physical changes in morphology and cytoskeletal organization [6]. These phenotypic changes are accompanied by a reduction in the expression of epithelial genes, such as cytokeratins, E-cadherin, and zona occludens (ZO)-1 and a gain in the expression of mesenchymal genes, such as vimentin, α-smooth muscle actin (αSMA), N-cadherin, fibronectin, and collagen-I [7]. EMT is regulated by the cellular microenvironment and can be induced by chemical and physical stimuli and maintained through epigenetic reprogramming. EMT is not a binary event, but rather it is a dynamic and reversible process, often termed epithelial–mesenchymal (E/M) plasticity [8], which allows for cancer cells to acquire a diversity of phenotypes and contribute to intratumoral heterogeneity.

Intratumoral heterogeneity complicates therapeutic decisions and contributes to unsuccessful treatments in cancer patients. The cooperative role that epigenetic reprogramming, EMT, and the tumor microenvironment play in promoting intratumoral heterogeneity is not well understood; addressing this knowledge gap will provide opportunities to improve therapeutic practices and outcomes for cancer patients. This review will discuss how E/M plasticity promotes tumor heterogeneity and the role that epigenetic reprogramming plays in maintaining hybrid E/M phenotypes in tumors. This review will also describe how the diverse mechanical properties across the tumor landscape regulate E/M plasticity and epigenetic factors. Furthermore, this work will describe how cellular diversity in tumors impacts cancer treatments and provide insights into how events that contribute to tumor heterogeneity can be targeted to overcome therapeutic challenges.

## 2. Epithelial–Mesenchymal Plasticity Contributes to Tumor Heterogeneity

**Figure 1:**
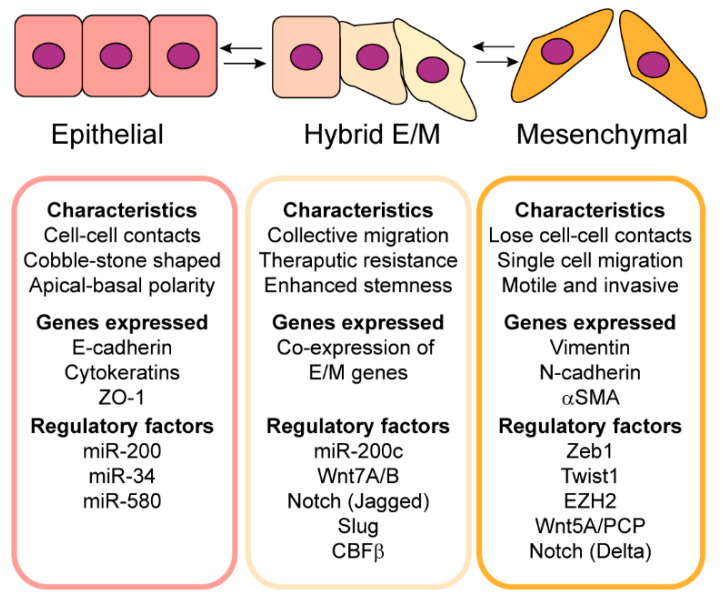
Cancer cells can exhibit epithelial–mesenchymal plasticity, which contributes to phenotypic heterogeneity within tumors.

Tumor cells can exhibit E/M plasticity, resulting in phenotypes along a spectrum ranging from epithelial to mesenchymal as well as intermediate E/M states that co-express epithelial and mesenchymal markers, and this contributes to phenotypic diversity within the tumor (Figure 1). Indeed, intermediate E/M phenotypes have been observed in multiple types of cancer and have been associated with poor prognosis. For example, keratin and vimentin are co-expressed in individual malignant mesothelioma and metastatic adenocarcinoma cells obtained from the ascitic fluid from human patients [9]. In the context of ovarian cancer, single-cell phenotypic characterization using multiparametric mass cytometry of high-grade serous ovarian cancer cells identified a subpopulation of hybrid cells that co-express E-cadherin and vimentin, and this hybrid phenotype correlates with a metastatic trajectory in patients that experience relapse [10]. Compared to epithelial and mesenchymal phenotypes, hybrid cells show increased levels of stem cell markers (CD24, CD49f, CD133, ROR, and β-catenin), genes related to a dysregulated cell cycle (pRb, cyclin B1, and pS6), and the metastasis promoting gene CD151 [10]. Furthermore, in a mouse model of prostate cancer, a subpopulation of tumor cells exhibit a hybrid E/M phenotype, indicated by co-expression of epithelial cell adhesion molecule (Epcam) and vimentin [11]. Compared to epithelial and mesenchymal phenotypes, the hybrid E/M cells form significantly more tumor spheres and have an increased percentage of stem/progenitor cells with a basal cell phenotype and the ability to initiate tumorigenesis, suggesting enhanced stemness [11]. Together, these studies illustrate that compared to epithelial or mesenchymal phenotypes, cancer cells that have hybrid E/M phenotypes have increased stemness and tumor-initiating potential.

The application of single-cell RNA sequencing has been instrumental in examining gene expression differences in cancer cells within a tumor. A recent study compared single-cell and bulk RNA sequencing data from a variety of tumors and determined that the expression of EMT signature genes in a tumor can reflect the presence of cancer-associated fibroblasts and cancer cells with partial EMT phenotypes [12]. This study identified laminins (LAMC1, LAMC3, and LAMA3), integrins (ITGA2 and ITGB1), CD44, and PVR as the top partial EMT-associated genes; however, there was significant variability in markers across squamous-like, gastro-intestinal, and gynecological cancers. In another study, single-cell RNA-sequencing revealed that a cluster of breast cancer cells from mammoplasty samples with aldehyde dehydrogenase activity (ALDH^+^) express high levels of the epithelial genes KRT7, KRT8, EPCAM, and CDH1 and high levels of the mesenchymal genes IL6, CD44, TM4SF1, and VIM [13]. In addition, these cells have high expression of breast stemness markers and they express genes related to aggressive triple-negative breast cancers [13]. Together, these studies demonstrate that the expression of EMT-associated genes can vary considerably between different types of cancer and between cells within the same tumor. As such, it is challenging to define a single EMT signature for the classification of cancer cells.

**Figure 2:**
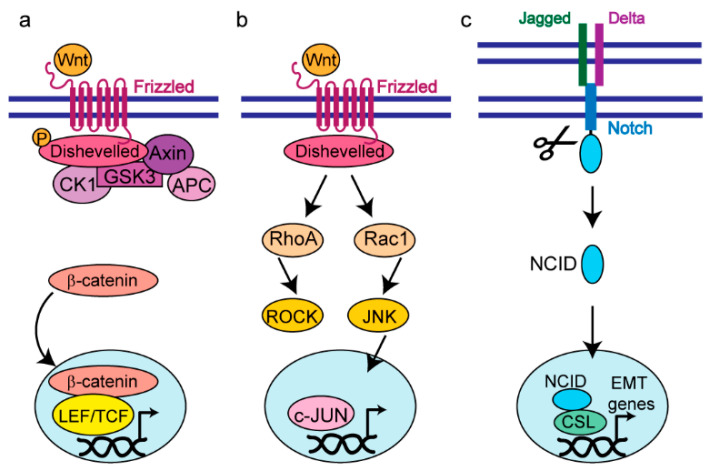
Signaling pathways involved in the regulation of E/M plasticity and gene expression. (**a**) Canonical Wnt signaling where Wnt binds to Frizzled to activate Dishevelled. Dishevelled interacts with components of the β-catenin destruction complex and β-catenin can translocate to the nucleus. In the nucleus, β-catenin binds to the lymphoid enhancer factor/T cell factor (LEF/TCF) complex to regulate gene expression. (**b**) In non-canonical Wnt signaling, small GTPases Rac and RhoA are activated, which leads to the activation of JUN N-terminal kinase (JNK) to regulate gene expression. (**c**) Notch signaling is activated when a Notch ligand, either Jagged or Delta, binds to a Notch receptor, promoting the cleavage and release of the Notch Intracellular Domain (NCID). The NCID translocates to the nucleus and binds to C protein binding factor 1/Suppressor of Hairless/Lag 1 (CSL) to regulate EMT gene expression.

Studies have provided mechanistic insight into the signaling pathways that control the maintenance of hybrid E/M phenotypes in cancer cells, and several of these pathways are highlighted in Figure 2. For example, tumors formed from hybrid E/M human breast cancer cells injected into mouse mammary fat pads were 10-fold larger and had a significantly higher frequency of tumor-initiating cancer stem cells than tumors formed from injected epithelial or mesenchymal cells, suggesting that a hybrid E/M state, rather than a mesenchymal or epithelial state, promotes tumorigenicity [14]. In this system, maintenance of the E/M state requires canonical Wnt signaling via Wnt7A/B, and transition to a mesenchymal state downregulates canonical Wnt signaling, upregulates noncanonical Wnt signaling via Wnt5A/planar cell polarity (PCP) signaling, and reduces tumorigenicity [14].

Notch signaling, which facilitates cell–cell and cell–matrix communication, has also been found to be a critical regulator of E/M maintenance [15]. A mathematical model predicted that in the presence of an EMT inducer, such as transforming growth factor (TGF)-β1, Jagged and Delta, two ligands that mediate Notch signaling, are activated and can induce a partial or complete EMT. When most cells exhibit a complete EMT phenotype, signaling is dominated by Delta while signaling dominated by Jagged promotes stabilized clusters of hybrid E/M cells, suggesting that Jagged, but not Delta, activation can induce a stable E/M phenotype. These predictions were supported experimentally by showing that breast cancer cells with hybrid E/M phenotypes, marked by co-expression of CD44 (mesenchymal mark) and CD24 (epithelial mark), have increased levels of cleaved Notch intercellular domain (NICD), which activates Jagged but represses Delta, compared to mesenchymal cells [15]. Another study utilized single-cell RNA-sequencing of MCF10A cells during TGFβ1-induced EMT to reveal that individual cells within a population undergo EMT at different rates [16]. After 8 days of TGFβ1 exposure, there was a significant gain in mesenchymal features such as increased CDH2, FN, and SNAI2 expression and activation of NOTCH and Wnt signaling, suggesting a shift toward a mesenchymal phenotype across the entire population. However, about half of the cells stably co-expressed epithelial and mesenchymal genes, suggestive of a hybrid E/M state. Furthermore, gene signatures that correspond to the E/M hybrid state are associated with poor patient outcomes [16].

Transcription factors including Snail, Slug, and Twist are considered master regulators of EMT and are associated with tumor aggressiveness and poor patient prognosis. Snail and Slug, which belong to the same family of zinc-finger transcription factors, are differentially expressed in mammary tumors and examination of mammary lesions has revealed that Snail is associated with the acquisition of a more complete mesenchymal phenotype [17]. Indeed, a recent study demonstrated that Snail acts as a stronger inducer of EMT in prostate cancer and mammary epithelial cells than Slug [18]. Mathematical modeling, which incorporated interconnected signaling between Snail and Slug, revealed that Slug promotes the maintenance of a hybrid E/M phenotype. These findings suggest that Slug can serve as a phenotypic stability factor [18]. The dysregulation of the Runt-related transcription factor (RUNX) family has also been implicated in the induction of EMT in the context of cancer, and RUNX2 has been shown to be a positive regulator of Slug [19]. ATAC-seq from subpopulations of SUM149PT breast cancer cells that exhibit hybrid E/M phenotypes shows enrichment for motifs of the RUNX family [20]. Knockdown of RUNX2 in cells with hybrid E/M phenotypes did not alter EMT markers; however, knockdown of core binding factor β (CBFβ), a coactivator of RUNX2, promoted downregulation of mesenchymal markers and acquisition of a more epithelial phenotype [20]. Further studies are required to determine whether CBFβ coordinates with Slug to stabilize hybrid E/M states.

While the abovementioned studies demonstrate that Wnt, Notch, and transcription factor signaling mediate E/M maintenance, a complete understanding of the molecular mechanisms controlling phenotypic transitions and maintenance of hybrid E/M states is lacking. A mathematical model was recently developed to predict how perturbations to gene regulatory networks impact E/M plasticity [21], but further studies are needed to examine how microenvironmental signals including tumor mechanical properties and heterotypic cell–cell interactions between cancer cells and other types of cells such as fibroblasts and immune cells that are found within the tumor microenvironment contribute to the acquisition and maintenance of E/M states.

## 3. Epigenetic Regulation of Epithelial–Mesenchymal Plasticity and Tumor Heterogeneity

Factors such as chromatin remodeling, histone modifications, and DNA methylation have been identified as regulators of cell phenotype and may contribute to the acquisition and maintenance of epithelial, mesenchymal, and E/M states within tumor cells. DNA is packed within chromatin, a structure that is ever-changing and can be influenced by external cues such as the rapidly changing chemical and physical properties of the tumor microenvironment. Post-translational modifications to histones, proteins that are components of chromatin, are key regulators of chromatin structure. Each histone has an amino acid tail that is susceptible to modification, such as the deposition or removal of functional groups. These modifications control chromatin structure by enhancing or disrupting the affinity of negatively charged DNA to the histone tail, thereby tightening or loosening chromatin and impacting DNA accessibility [22]. Histone modifications and their modifying enzymes also recruit protein complexes to impact chromatin reorganization [23]. Chromatin remodeling is critical to maintaining normal cellular processes, but the misregulation of histone-modifying enzymes and chromatin remodeling have been linked to cancer [24,25,26,27].

A mouse model of skin squamous cell carcinoma identified subpopulations of cells that undergo EMT at different rates and have distinct chromatin landscapes [28]. The subpopulations include mainly epithelial (Epcam^+^), mainly mesenchymal (Epcam^−^), and hybrid E/M phenotypes that were characterized based on the expression of the tumor stemness-associated genes CD51, CD61, and CD106. The subpopulations differentially expressed the epithelial marker keratin 14 and the mesenchymal marker vimentin. In the CD51^−^/CD61^−^/CD106^−^ subpopulation, 5% of cells expressed keratin 14, 15% expressed vimentin, and 80% of cells co-expressed keratin 14 and vimentin and the CD51^−^/CD61^−^/CD106^−^ subpopulation exhibited closed chromatin at the Epcam and CDH1 regions and open chromatin at the KRT14 and KRT17 regions. In the CD106^+^ subpopulation, about 20% of cells expressed vimentin and 80% co-expressed keratin 14 and vimentin. In the CD51^+^/CD61^+^/CD106^+^ subpopulation, 90% of cells expressed vimentin and 10% co-expressed keratin 14 and vimentin. The more mesenchymal subpopulations had open chromatin regions at the vimentin, Zeb1, Aspn, and col24a1 promoter regions. Furthermore, clustering analysis of ATAC-seq data revealed that chromatin remodeling in these subpopulations falls into three distinct clusters that are associated with the different states of EMT. Cluster 1 includes the more epithelial subpopulations Epcam^+^ and CD51^−^/CD61^−^/CD106^−^, cluster 2 includes the hybrid subpopulations CD61^+^ and CD106^+^/CD51^+^, and cluster 3 includes the more mesenchymal subpopulations CD51^+^/CD61^+^ and CD51^+^/CD61^+^/CD106^+^. These subpopulations were found to localize to four distinct regions of squamous cell carcinoma tumors. The more epithelial region showed high keratin 14 and low vimentin expression, while the cells in the hybrid region were elongated, maintained cell–cell contacts, and co-expressed keratin 14 and vimentin. In another region, most of the cells had undergone EMT; cells were elongated with low cell–cell adhesion, low keratin 14 expression, high vimentin, and most of the cells expressed CD61. In the final region, all cells had completely undergone EMT indicated by elongation, no cell–cell adhesion, no keratin 14 expression, and high expression of vimentin and CD61 [28]. These findings highlight that subpopulations of tumor cells undergo EMT at different rates, have distinct chromatin landscapes, and have differential spatial localization within tumors.

DNA methylation, which has been shown to increase chromatin condensation and promote a heterochromatin state [29], is essential for normal cell function but is misregulated in cancer. Indeed, hypermethylation at the promoter regions of tumor suppressor genes promotes silencing and can drive tumorigenesis [30,31,32]. For example, increased DNA methylation and intratumor heterogeneity are associated with larger tumor size and increased risk of relapse post-surgery in lung adenocarcinoma patients [33]. DNA hypermethylation at the promoters of five tumor suppressor genes (RAAF1A, p16, DAPK, MGMT, and Rb) was examined in melanoma tumors, and 70% of the tumors had heterogeneous methylation patterns with methylation patterns of samples from the tumor core being more representative of the entire tumor than samples at the tumor edge [34]. Hypoxia, which can occur at the tumor core, is associated with reduced activity of ten-eleven translocation (TET) methylcytosine dioxygenases, which regulate the de-methylation of DNA, resulting in increased DNA methylation of tumor promoter genes [35]. Furthermore, cancer cells at the tumor edge have been found to exhibit E/M plasticity and a migratory phenotype [36,37]; it is possible that differences in DNA methylation patterns of epithelial genes in cells found within different regions of the tumor contribute to differences in E/M phenotypes within tumor cells. Further studies are needed to determine how factors such as oxygen tension and mechanical properties, which vary spatially within tumors, impact DNA methylation and the expression of epithelial and mesenchymal genes.

DNA methylation has been found to regulate transcription factors that contribute to E/M maintenance in cancer cells. H1299 lung adenocarcinoma cells exhibit a mesenchymal phenotype, marked by high vimentin expression levels and no E-cadherin expression, and these cells migrate as individual cells; in contrast, H1975 lung adenocarcinoma cells express both vimentin and E-cadherin, indicating an E/M phenotype, and these cells migrate collectively [38]. Knockdown of the DNA binding transcription factors Grainyhead-like 2 (GRHL2) and Ovo Like Zinc Finger 2 (OVOL2) in H1975 cells destabilizes the hybrid E/M phenotype and induces EMT, marked by loss of E-cadherin and gain of Zeb1 [38]. Knockdown of GRHL2 and OVOL2 also enhances individual cell migration. In support of these findings, a mathematical model predicts that GRHL2 stabilizes the hybrid E/M phenotype and promotes tumor-initiating properties [38]. GRHL2 and OVOL2 contribute to E/M maintenance by coupling to the miR-200/ZEB circuit and forming mutually inhibitory loops with ZEB. Indeed, DNA (cytosine-5)-methyltransferase 3A (DNMT3A) represses GRHL2 transcription by hypermethylating the GRHL2 promoter, resulting in ZEB activation and EMT induction, and knockdown of DNMT3A blocks EMT [39]. Similarly, the OVOL2 promoter is hypermethylated in late-stage colorectal cancer cells, resulting in OVOL2 suppression [40]. Furthermore, nasopharyngeal carcinoma cells have decreased levels of OVOL2 and E-cadherin and increased levels of N-cadherin compared to normal nasopharyngeal epithelial cells, and the low levels of OVOL2 are a result of hypermethylation at the promoter region [41]. High levels of OVOL2, GRHL2, and CDH3, which are markers for an E/M hybrid phenotype, correlate with poor survival in bladder, breast, liver, and colon cancer patients [38]. These studies suggest that DNA methylation mediates the suppression or expression of transcription factors, and these factors coordinate to regulate the maintenance of the E/M phenotype.

Aberrant expression and activation of epigenetic modifiers are present in multiple types of cancers and are associated with the misregulation of genes implicated in cancer metastasis. The addition of acetyl groups to histones, which is regulated by histone acetyltransferases and histone deacetylases (HDACs), alters chromatin structure and gene transcription. Indeed, HDAC 1, 2, and 3 are overexpressed in lung, gastric, liver, and bladder cancer [42,43,44,45]. Misregulated expression or activation of HDACs can result in the repression of tumor suppressor genes or the activation of oncogenes. For example, the oncogenic fusion protein complex acute myeloid leukemia/RUNX1T1 (AML1-ETO) aberrantly recruits HDAC 1 to AML1 target genes to promote the progression of acute myeloid leukemia [46]. Histone deacetylation also plays a role in regulating E/M plasticity. For example, the deacetylation of H3K56 at the E-cadherin promoter suppresses E-cadherin expression in prostate cancer [47]. Histone deacetylation may play a role in promoting E/M phenotypes; in breast cancer cells, the inhibition of histone deacetylation promotes hybrid E/M phenotypes indicated by the co-expression of E-cadherin and N-cadherin [48].

Histone methylation, which is regulated by histone methyltransferases and demethylases, is also misregulated in cancer and associated with altered E/M gene expression. For example, the enhancer of zeste homolog 2 (EZH2) is a methyltransferase that tri-methylates histone 3 lysine 27 (H3K27me3), and EZH2 is overexpressed in multiple types of cancer [24,25,49,50,51]. In addition, EZH2 serves as an activator and suppressor of genes associated with EMT and apoptosis. EZH2 suppresses apoptosis of prostate cancer cells by silencing the pro-apoptotic microRNA miR-31 via trimethylation of H3K27 at the miR-31 gene promoter region [52]. EZH2 and H3K27me3 levels are also increased at the E-cadherin gene promoter in prostate and pancreatic cancer cells, resulting in the suppression of E-cadherin and promotion of EMT [53,54]. Moreover, EZH2 can also act as an activator of genes to drive EMT; EZH2 promotes the expression of the mesenchymal marker αSMA by forming a transcriptional complex with Smad2 and binding to the ACTA2 gene promoter in atrial fibroblasts [55]. Heterogeneous expression and subcellular localization of EZH2 in tumors have also been linked to invasion and poor prognosis. Single-cell RNA-seq of patient-derived glioblastoma cells revealed the presence of a subpopulation of cells with high EZH2 expression that correlated with poor patient survival [56]. In another study, immunohistochemical staining showed that in melanoma cells, the levels of EZH2 and H3K27me3 are increased in cells at the invasive front compared to cells found in the center of the tumor [57]. Furthermore, phosphorylation of EZH2 can mediate its subcellular localization and function, and cytoplasmic pEZH2-T367 enhances breast cancer invasion; in breast cancer cells, p38 phosphorylates EZH2 at T367 to induce EZH2 cytoplasmic localization and binding to vinculin, thereby promoting invasion [58]. In metaplastic breast cancer tissue samples, immunohistochemical staining revealed that cytoplasmic pEZH2-T367 is significantly higher in cells located at the tumor periphery compared to cells located at the tumor center and that cytoplasmic EZH2 interacts with actin-binding proteins and proteins involved with cell migration (MYO1B, MYO1D, MYO1F, DBNL, and TLN1) [59]. Together, these studies demonstrate the diversity of EZH2 expression and localization across tumors. EZH2 is known to regulate genes involved in E/M plasticity, and thus EZH2 may contribute to the differences in E/M phenotypes observed in cancer cells as a function of location within the tumor.

Epigenetic factors can also regulate tumor cell proliferation and tumor growth. In triple-negative breast cancer cells, there is a subpopulation of cells with high EZH2 expression that exhibit increased mammosphere formation and metastatic potential, and inhibition of EZH2 reduces mammosphere size and abundance [60]. High nuclear levels of EZH2 correlate with increased proliferation, indicated by high Ki67 expression, and poor prognosis in melanoma, prostate, endometrial, and breast cancer [61]. Indeed, EZH2 inhibition reduces proliferation in melanoma cells and reduces tumor growth in a mouse model of melanoma [62]. Another study demonstrated that JARID1B, a H3K4 demethylase that is expressed in a small subpopulation (5–10%) of the total cell population in aggressive primary and metastatic melanomas, may promote tumor maintenance by controlling cell proliferation [63]. Cells expressing JARID1B have low expression of the proliferation marker Ki67 and a low doubling rate and are not actively proliferating. Knockdown of JARID1B revealed that JARID1B is required for continuous tumor growth and progression. These results suggest that JARID1B-expressing cells form a slow-cycling subpopulation that exhibits high self-renewal that contributes to melanoma tumor maintenance [63]. The abovementioned studies demonstrate that methylation of histone marks within tumor cells is heterogeneous and that histone methylation patterns contribute to tumor maintenance by regulating the proliferation and growth of cancer cells. Future studies examining the variation in other histone marks and histone-modifying enzymes within tumors may provide new insights into differential gene expression and growth properties within tumors.

Signaling molecules such as microRNAs and transcription factors interface with epigenetic pathways to control the induction of EMT and maintenance of diverse phenotypes within tumors. Indeed, studies have demonstrated that a miR-200/ZEB/miR-34/SNAIL circuit regulates EMT, with high ZEB/SNAIL expression mediating the acquisition of mesenchymal features while high miR-200/miR-34 expression maintains an epithelial state [64,65,66]. For example, exogenous expression of miR-200 in cancer cell lines promotes the expression of E-cadherin while inhibition of miR-200 promotes the acquisition of a mesenchymal phenotype with reduced E-cadherin expression and increased vimentin expression [64]. Furthermore, ectopic expression of miR-200c in H460 lung cancer cells increases the percentage of hybrid E/M cells from ~5% to ~20% and these hybrid cells have increased responsiveness to external stimuli such as TGFβ1 [67]. To examine whether a phenotypic state can be stabilized in the context of EMT, epigenetic feedback was incorporated within a mathematical model focused on the regulation of E/M plasticity by the miR-200/ZEB/miR-34/SNAIL circuit [66]. These studies found that high Zeb1 levels present in mesenchymal cells can inhibit miR-200, which can stabilize the mesenchymal phenotype by promoting long-term transcriptional activity [66]. To confirm these predictions, experiments were performed with MCF10A cells to examine the impact of cellular memory on EMT progression and reversion. MCF10A cells exposed to TGFβ for short timescales (3–6 days) undergo EMT and can revert to an epithelial phenotype upon removal of TGFβ. In contrast, cells exposed to TGFβ for longer durations (12–15 days) maintained a mesenchymal phenotype even after TGFβ removal for an additional 15 days [66]. A follow-up study found that MCF10A cells exposed to TGFβ1 for long durations (22 days) could revert to an epithelial phenotype 45 days after TGFβ1 withdrawal and that the delayed reversion to the epithelial state is due to epigenetic repression of miR-200 by ZEB [68]. These findings suggest that epigenetic feedback may play an important role in the maintenance of E/M phenotypes, and that stabilization of cell phenotypes may shift the response of cells to chemical and physical cues from the tumor microenvironment. Further studies examining the impact of chemical and mechanical memory on E/M plasticity will provide a more detailed understanding of factors that regulate the stability of E/M states.

## 4. Regulation of Epithelial–Mesenchymal Plasticity and Epigenetics by Mechanical Heterogeneity in the Tumor Microenvironment

During cancer progression, cells undergo significant changes in proliferative state and phenotype, resulting in a complex tumor microenvironment that influences the mechanical properties of the tissue. Cancer cell proliferation escalates as the disease progresses, and the rapid increase in cell number within the tumor exerts a solid stress on the surrounding tissue [69]. Furthermore, extracellular matrix (ECM) remodeling due to changes in deposition and degradation of matrix components promotes changes in the tumor microenvironment; increased ECM deposition mediated by cancer-associated fibroblasts contributes to the increase in matrix stiffness that is observed in many cancers [70,71]. Indeed, cancer progression is accompanied by increased collagen density and alignment [72,73]. Increases in solid stresses can compress the blood and lymphatic vessels within the tumor, resulting in vessel leakage and changes in the tumor interstitial fluid pressure [69]. The mechanical properties of the ECM are also influenced by matrix metalloproteinases (MMPs), which contribute to matrix degradation and are important in EMT and invasion; MMP3 promotes EMT in mammary epithelial cells [74,75] and MMP7 induces early-stage tumorigenesis in mouse models [76]. Evidence supports that the significant alterations in the mechanical properties of the tumor microenvironment that occur as cancer progresses play an important role in regulating cell phenotypic changes, thus contributing to the heterogeneity observed in tumors.

**Figure 3:**
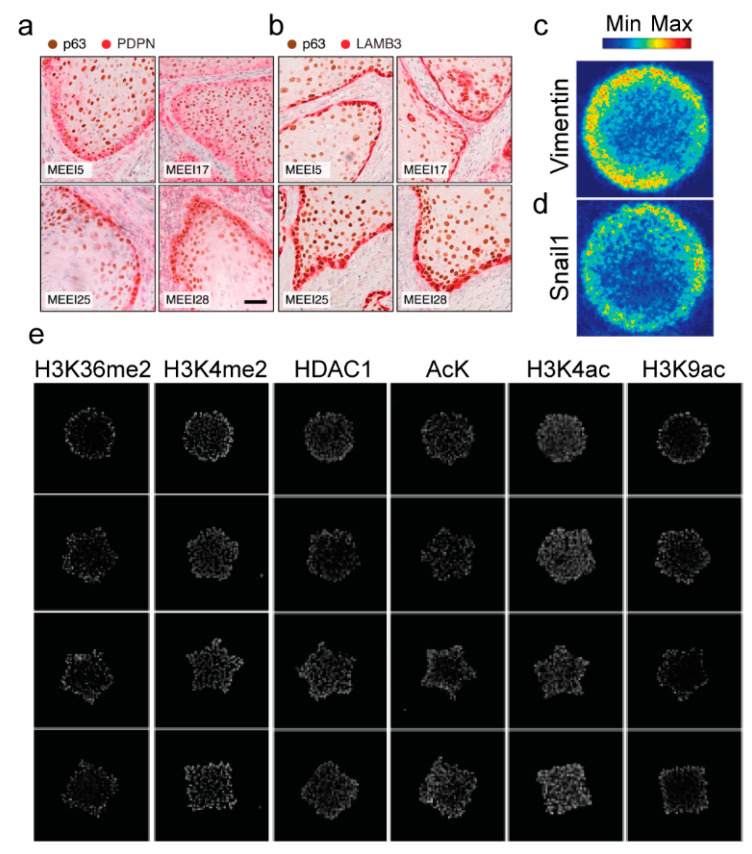
E/M phenotypes and epigenetics modifications vary as a function of spatial location within tissues and tumors. Immunohistochemical staining images of head and neck squamous cell carcinoma tumors (MEEI5, MEEI17, MEEI25, and MMEI28) stained for the p-EMT markers (**a**) PDPN and (**b**) LAMB3 (red) and the malignant cell-specific marker p63 (brown), Scale bar: 100 μm. Reprinted from *Cell* vol 171, Puram, S., et al., Single-cell transcriptomic analysis of primary and metastatic tumor ecosystems in head and neck cancer, 1611–1624 Copyright (2017), with permission from Elsevier [81]. (**c**) Heatmaps showing the frequency of (**c**) vimentin and (**d**) Snail1 expression in MCF7 breast cancer cells cultured on fibronectin-coated circular islands. Reprinted from *Biomaterials*, vol 281, Lin, F., et al., Spontaneous formation and spatial self-organization of mechanically induced mesenchymal-like cells within geometrically confined cancer cell monolayers, 121337, Copyright (2022), with permission from Elsevier [96]. (**e**) Immunofluorescence staining for the histone modification marks and modifying enzymes H3K36me2, H3K4me2, HDAC1, AcK, H3K4ac, and H3K9ac in B16F0 melanoma cells. Adapted with permission from Ref. [100]. 2020, Springer Nature.

The compositional and mechanical properties of the tumor microenvironment vary across the tumor landscape and promote tumor progression. For example, in PyMT mice, vasculature is significantly stiffer at the tumor core, while the ECM is significantly stiffer at the tumor-invasive front [77]. The spatial differences in matrix stiffness within tumors likely contribute to intratumoral phenotypic heterogeneity and E/M plasticity. Indeed, a recent study utilized a neural network to predict the elastic properties of tumor stroma from a combination of AFM and collagen morphology data and found that in human breast tumors, the expression of EMT markers colocalized with regions of the tumor that have high elasticity [78]. Moreover, increased extracellular matrix stiffness at the tumor periphery may facilitate the spread of cancer; EMT is more prominent at the invasive front of tumors than at the tumor core [37,79]. Furthermore, in multicellular mouse colon carcinoma spheroids, cells located at the periphery are more motile and proliferative than cells located at the center [80]. Another study determined that cells with a hybrid phenotype are localized to the edges of tumors [81]. Single-cell RNA-seq of cells from head and neck squamous cell carcinoma (HNSCC) primary tumors and matched metastatic lymph node metastases revealed a subpopulation of cells that co-express both epithelial (KRT14, KRT17, KRT5, and EPCAM) and mesenchymal (TGFB1, LAMC2, ITGA5, and VIM) genes, termed a partial EMT (p-EMT). Primary tumors with high p-EMT scores correlated with higher tumor grade, number of metastases, and higher nodal stage. HNSCC tumors were stained for the p-EMT markers PDPN, LAMB3, LAMB2, MMP10, TGFB1, ITGA5, and cells that co-stained for p-EMT markers localized to the leading edge of tumors (Figure 3a,b) and near cancer-associated fibroblasts, suggesting that the tumor microenvironment influences p-EMT [81]. Overall, these findings suggest that differences in chemical and physical properties found at the core and periphery of tumors may influence phenotypic diversity within tumors.

A number of in vitro studies have demonstrated that matrix mechanical properties regulate EMT. Culture of pancreatic cancer cells on stiff hydrogels mimicking the mechanical properties of tumors promotes higher expression of mesenchymal markers and a decrease in the expression of epithelial markers in comparison to culture on soft hydrogels [82]. For mammary epithelial cells, TGFβ1 treatment induces αSMA expression in a subpopulation of cells (~15%) when cultured on stiff 6300 Pa but not soft 300 Pa matrices, suggesting that cells within a population exhibit differential responses to TGFβ1 and matrix stiffness [83]. In addition, stiff microenvironments have been found to support EMT-associated multinucleation, a precursor to aneuploidy, in a small subset of mammary epithelial cells overexpressing Snail [84]. Dynamic modulation of matrix stiffness also regulates EMT; an increase in stiffness promotes a more mesenchymal phenotype in mammary epithelial cells [85] while softening of an initially stiff microenvironment promotes mesenchymal–epithelial plasticity, a reduction in proliferation, and an increase in apoptosis in breast cancer cells [86]. A large majority of studies examining the impact of matrix mechanics on EMT have focused on the role of matrix elasticity, and few studies have examined how matrix viscoelasticity regulates EMT. One recent study found that for mammary epithelial cells cultured on stiff matrices, TGFβ1-induces αSMA in a subpopulation of cells (~12%) when the matrix exhibits low viscous dissipation, while TGFβ1 induces apoptosis when the matrix exhibits high viscous dissipation [87]. Collectively, studies using 2D in vitro models have provided mechanistic insight into how matrix mechanics regulate E/M phenotypes and have revealed heterogeneity in cell response to EMT induction cues.

Recent efforts have focused on the development of 3D tumor models to examine the impact of matrix mechanics on E/M plasticity and cancer heterogeneity [88,89,90,91,92,93]. For example, mammary cells cultured in stiff matrices undergo EMT via a TWIST1-dependent mechanism, indicated by decreased E-cadherin and increased fibronectin expression while cells cultured in soft matrices retain an epithelial phenotype [88]. In another study, MCF7 cells cultured in matrices that soften from 2700 Pa to 500 Pa exhibit changes in the expression of E/M-associated proteins and have increased sensitivity to doxorubicin as the matrix softens [89]. Matrix adhesiveness also influences E/M plasticity in 3D; prostate cancer cells cultured in matrices with increased adhesiveness readily undergo EMT and invade the matrix [90]. Furthermore, the use of a 3D microfluidic tumor model demonstrated that cancer cells with epithelial and mesenchymal traits determine tumor cell growth and invasion, respectively, and that small numbers of mesenchymal-like cancer cells within a heterogeneous multiclonal tumor can drive overall tumor invasion [91]. The use of biosensors to detect single-cell gene expression with 3D models of cancer also offers the possibility to examine cellular heterogeneity during tumor invasion into the surrounding matrix. This approach has been demonstrated through the use of gold nanorod-locked nucleic acid (GNR-LNA) biosensors to examine Delta-like ligand 4 (DLL4) expression in a bladder cancer invasion model to reveal that DLL4 expression is upregulated at the invading front of tumor spheroids [92]. Further development of more sophisticated 3D tumor models that better recapitulate the tumor microenvironment and tools to examine single-cell gene expression will reveal new insights into mechanisms governing E/M plasticity and the impact of matrix mechanics on intratumor heterogeneity.

The maintenance of hybrid E/M states may also be influenced by the physical properties of the microenvironment. Mathematical modeling and experimental validation determined that Src overexpression reduces receptor-type tyrosine-protein phosphatase (RPTP) and promotes a transition from an epithelial to a mesenchymal phenotype, indicated by a decrease in E-cadherin and an increase in phosphorylated focal adhesion kinase (pFAK) [94]. Forced expression of RPTP in Src overexpressing cells induces a hybrid phenotype, marked by restoration of cell–cell contacts but high pFAK levels, in 50% of cases and a mesenchymal phenotype in 50%. When cells are cultured in a soft (~1600 Pa) ECM, overexpression of Src induces a hybrid phenotype, while in a stiff (~3000 Pa) ECM, Src overexpression induces a mesenchymal phenotype [94]. These studies demonstrate that matrix stiffness plays a critical role in regulating cell phenotype; therefore, the spatial and temporal differences in mechanical properties that are observed within tumors are likely to contribute to intratumoral heterogeneity.

Tissue and tumor geometry are also altered during cancer progression, which can induce mechanical stress gradients within tumors. Previous studies have shown that tissue-level mechanical stress impacts cell proliferation, EMT, cancer stem cell properties, histone modifications, and histone-modifying enzymes. The culture of mouse mammary cells in epithelial sheets with micropatterned geometry revealed that cells located in regions of the tissue that experience high mechanical stress, such as the edges and corners of square-shaped microtissues, undergo TGFβ1-induced EMT, while cells cultured in regions of low mechanical stress do not [95]. In another study, MCF7 cells cultured in micropatterned circular monolayers exhibit increased vimentin and Snail1 expression at the edges of the microtissues compared to the center (Figure 3c,d) [96]. Furthermore, branching morphogenesis, the process that occurs during mammary gland formation, is initiated by EMT at branch tips to enable invasion into the surrounding ECM. Normal mouse mammary epithelial cells cultured in 3D microtubules undergo branching in areas of the tubule that experience the highest mechanical stress and have elevated vimentin gene promoter activity in cells located where branching occurs [97]. Tissue level stresses also impact cell proliferation. Endothelial cells cultured within geometrically patterned sheets exhibit increased proliferation of cells located at regions that experience high mechanical stress [98]. Whether this is also true for the proliferation of cancer cells is not clear. In addition, tissue geometry regulates cancer stem cell properties; the expression of cancer stem cell-associated genes in malignant melanoma cells is increased at the periphery of micropatterned tissues [99]. B16F0 murine melanoma cells cultured within micropatterned tissues of various shapes (corresponding to different patterns and gradients of mechanical stress) have higher levels of H3K4me2, H3K36me2, HDAC1, H3K4ac, H3K9ac, and global lysine acetylation at the periphery of tissues compared to the center (Figure 3e). The expression of PRDM14, an epigenetic modifier linked to cancer severity, is also increased at the perimeter compared to the center of micropatterned tissues. PRDM14 knockdown attenuates the acquisition of the malignant phenotype [100]. These results suggest that the higher mechanical stress experienced at the edges of micropatterned tissues may promote epigenetic reprogramming thereby contributing to melanoma malignancy. Furthermore, these findings suggest that E/M plasticity occurrence at the edges of tumors and acquisition of cancer stem cell properties may be mediated in part by heterogeneities in mechanical stress within tumors.

The previously mentioned studies have highlighted that the phenotypes of cells within tumors are diverse and can range from epithelial to mesenchymal with intermediate states. These E/M phenotypes can be induced and maintained by epigenetic factors and the mechanical properties of tumor tissue. Indeed, E/M plasticity in tumor cells has been shown to promote drug resistance and tumor recurrence. The following section will describe mechanisms that contribute to therapeutic resistance and discuss potential targets to improve treatment efficacy.

## 5. Therapeutic Approaches to Target Tumor Cell Heterogeneity and Tumor Recurrence

Intratumoral heterogeneity makes therapeutic decisions difficult and poses challenges for successful treatments. E/M plasticity has also been shown to contribute to tumor recurrence; tumor cells that disseminate from primary tumors can acquire dormant phenotypes at distant sites that can promote late-stage tumor recurrence and resistance to therapy [101]. Furthermore, early disseminated cancer cells (eDCCs) are cancer cells that disseminate from the primary tumor site before a tumor is detectable and one study found that Her2^+^ breast eDCCs exhibit a hybrid E/M phenotype, marked by decreased E-cadherin and high cytokeratin 8 and 18 (epithelial marks) expression and increased Twist1 and invasion [102]. Most of the eDCCs examined in this study were non-proliferative, indicated by the low presence of p-retinoblastoma protein and low p-serine 10, but the cells were able to initiate metastasis, suggesting that this subpopulation of cells is capable of forming metastases after being in a dormant state [102]. Indeed, tumor cell dormancy promotes therapeutic resistance and tumor recurrence. Another study demonstrated that E/M plasticity controls the transition from dormant to proliferative in cancer cells at distant metastases [103]. MCF7 and T47D breast cancer cells, which are estrogen receptor-positive (ER^+^), were injected into the milk ducts of mice to generate ER^+^ breast cancer models. In ER^+^ models, distant lesions had significantly decreased proliferation (marked by low Ki67) and increased dormancy (marked by high p27) compared to primary tumors. Furthermore, compared to primary sites, in ER^+^ models, distant sites had decreased E-cadherin and Snail1, increased Zeb1 and vimentin, and similar Snail2 and Twist1 levels. Ectopic E-cadherin expression significantly increased the proliferation of cells at distant sites, suggesting that the transition to a more epithelial phenotype promotes the reawakening of dormant cells at distant sites [103]. Given the connection between E/M plasticity and tumor recurrence, an improved understanding of the signaling pathways that regulate E/M states could suggest new therapeutic approaches for treating and eradicating cancer.

In addition to cell phenotypic heterogeneity reducing therapeutic efficacy, variations in the mechanical environment of tumors also pose challenges to successful cancer treatments. There are physical barriers that impact drug delivery to the tumor. For example, drugs enter high collagen areas of tumors slower than low collagen areas [104,105]. In addition, the increased interstitial fluid pressure and leaky vessels that result from increased solid stresses within the tumor make drug delivery through vessels difficult [69]. Conversely, the leaky vasculature present in tumors can promote the accumulation of molecules within the tumor [106,107] and can be exploited using nanomedicine to improve drug delivery [108,109]. There is also evidence that mechanical properties of the cellular microenvironment independently mediate drug response in cells. For example, matrix stiffness can regulate response to therapeutics in different cell types; BxPC-3 pancreatic cancer cells cultured on 4 and 25 kPa polyacrylamide hydrogels have increased chemoresistance to paclitaxel compared to cells cultured on 1 kPa matrices, while resistance to gemcitabine is not influenced by matrix stiffness [82]. Another study identified integrin-linked kinase (ILK) as a signaling molecule that may control stiffness-mediated doxorubicin resistance in breast cancer cells [110]. MDA-MB-231 breast cancer cells were cultured on polyacrylamide hydrogels with low (10 kPa), intermediate (38 kPa), or high (57 kPa) Young’s moduli, and following treatment with doxorubicin, cells cultured on 10 and 57 kPa hydrogels exhibited higher apoptosis, higher uptake of doxorubicin, and lower proliferation compared to cells cultured on 38 kPa hydrogels. Cells cultured on 38 kPa hydrogels, compared to 10 and 57 kPa hydrogels, showed increased doxorubicin resistance, increased ILK RNA and protein levels, and increased YAP nuclear localization and activity. Inhibition of ILK reduced YAP nuclear localization and activity and attenuated doxorubicin resistance in cells cultured on the 38 kPa substrate [110]. Another study found that treatment with Tamoxifen reduces the proliferation of MCF7 breast cancer cells cultured on stiff 100 kPa matrices but does not impact the proliferation of cells cultured on soft 0.1 kPa matrices, and it was determined that the culture of cells on soft matrices promotes chemoresistance by inducing autophagy, which is mediated by ILK [111]. Microenvironmental factors, such as ECM stiffness, influence E/M plasticity and drug efficacy and thus could be a promising target for improving treatment outcomes. However, the cellular processes and signaling pathways that become aberrantly regulated in response to changes in ECM mechanics are not well understood and further research is needed to elucidate mechanistically how the misregulation of cell signaling occurs in order to determine therapeutic targets.

Epigenetic factors also contribute to therapeutic resistance. For example, inhibiting the activity of the histone lysine demethylase KDM5 reduces transcriptomic heterogeneity and reduces resistance to the chemotherapeutic fulvestrant in MCF7 breast cancer cells [112]. Furthermore, drug-tolerant glioblastoma stem cells have increased expression of the histone demethylase KDM6A/B and the corresponding modification H3K27ac and decreased EZH2 and H3K27me3 levels compared to naïve glioblastoma stem cells, and knockdown of KDM6A or KDM6B significantly reduces the emergence of drug-tolerant cells [113]. Another study found that reducing H3K27me3 levels via the inhibition of EZH2 activity in MDA-MB-468, BT20, and HCC38 triple-negative breast cancer cells promotes a drug-tolerant state, and treatment with a KDM6A/B inhibitor upon chemotherapy treatment reduces the number of drug-resistant cells and delays tumor recurrence in mouse models [114]. Thus, epigenetic regulation of chromatin states controls switches in cell phenotype to contribute to tumor heterogeneity and drug tolerance. While the correlation between epigenetic factors and drug tolerance is evident, how cancer cells utilize epigenetic machinery to exist in a drug-tolerant state is not well studied. Epigenetic modifications are reversible and thus provide an opportunity for therapeutic intervention. Indeed, pharmacological inhibition of EZH2 has been shown to reduce breast cancer metastasis [60,115] and enhance the efficacy of chemotherapeutics [116].

Several epigenetic drugs (epi-drugs) that inhibit DNA methyltransferases or histone deacetylases have shown success in clinical trials for the treatment of hematological malignancies [117,118,119]; however, efficacy for the treatment of solid tumors has been more limited (reviewed by [120,121]). Epi-drugs used in combination with other treatments such as chemotherapy, hormone therapy, and immunotherapy hold promise for improved efficacy. For example, treatment with the histone deacetylase inhibitor Tucidinostat (also known as Chidamide) in combination with the hormone treatment Exemestane significantly improved progression-free survival in advanced hormone receptor-positive breast cancer patients [122] but did not impact overall survival over a 6-year study [123]. In vitro, Chidamide treatment has been shown to attenuate TGFβ1-induced loss of E-cadherin expression in lung cancer cells [124]. Treatment with Etinostat, an HDAC inhibitor, reduced vimentin and N-cadherin expression and rescued E-cadherin expression in MDA-MB-231 breast cancer cells [125]. A phase 2 clinical trial showed that combination treatment with Etinostat and Exemestane significantly improved progression-free survival and overall survival in hormone receptor-positive breast cancer patients [126], but a follow-up phase 3 clinical trial found that this combination therapy did not impact overall survival [127]. Treatment with the HDAC inhibitor, Vorinostat, attenuates TGFβ1-induced loss in E-cadherin and gain in N-cadherin and vimentin in biliary tract cancer cells [128]. Encouraging clinical trial results have been obtained for the treatment of head and neck squamous cell carcinoma patients with Vorinostat in combination with chemoradiation therapy [129]. The study is ongoing, and 96.2% of patients have no detectable cancer following therapy, the estimated 5-year overall survival rate is 68.4%, and the estimated 5-year disease-free survival rate is 76.6% [129]. Heterogeneity within tumors is one factor that could limit the overall response to epi-drugs. As such, multi-region biopsy sampling combined with single-cell analyses to characterize cancer cell heterogeneity may help to predict how patients will respond to epi-drugs and could aid in patient cohort selection based on the presence of sensitizing alterations within the tumor [120]. While targeting epigenetic modifiers in cancer treatment has shown promising results, a better understanding of how these factors respond to variation in the tumor microenvironment and contribute to E/M plasticity, malignancy, and drug resistance is needed to improve cancer treatment and patient outcomes.

## 6. Conclusions and Future Perspectives

Intratumoral heterogeneity contributes to cancer severity, correlates with poor patient prognosis, and impedes the selection and efficacy of therapeutics. Improved methods for the detection and characterization of E/M phenotypes in tumors could better inform therapeutic decisions and enhance treatment efficacy. In order to improve patient outcomes, a better understanding of the molecular mechanisms that contribute to heterogeneity in the tumor microenvironment is needed. Epigenetic modifications can contribute to drug resistance in cancer cells, but research focused on elucidating how cancer cells utilize this reversible reprogramming to evade treatment is lacking. Efforts should be made to determine how cancer cells employ epigenetic programs to acquire phenotypes that are drug-resistant. An improved understanding of how cancer cells avoid treatments will provide opportunities for therapeutic intervention.

During cancer progression, the tumor microenvironment experiences significant alterations in mechanical properties that promote phenotypic changes in cancer cells. Epigenetic reprogramming regulates the maintenance of E/M plasticity and has been shown to be regulated by extracellular mechanical signals. It is evident that epigenetic modifications, E/M plasticity, and the tumor microenvironment impact tumor composition, but it is not clear how these factors cooperate to promote tumor heterogeneity. To address this knowledge gap, future research should focus on further elucidating the signaling pathways that mediate epigenetic modifiers that control gene expression and phenotypic switches in response to the tumor microenvironment. Improved understanding of how the chemical and mechanical properties of the tumor microenvironment control phenotypic plasticity will enable the identification of novel therapeutic targets and the development of treatments to improve cancer patient outcomes.
